# Identifying SARS-CoV-2 infected cells with scVDN

**DOI:** 10.3389/fmicb.2023.1236653

**Published:** 2023-07-10

**Authors:** Huan Hu, Zhen Feng, Xinghao Steven Shuai, Jie Lyu, Xiang Li, Hai Lin, Jianwei Shuai

**Affiliations:** ^1^Department of Physics, and Fujian Provincial Key Laboratory for Soft Functional Materials Research, Xiamen University, Xiamen, China; ^2^Wenzhou Institute and Wenzhou Key Laboratory of Biophysics, University of Chinese Academy of Sciences, Wenzhou, China; ^3^National Institute for Data Science in Health and Medicine, and State Key Laboratory of Cellular Stress Biology, Innovation Center for Cell Signaling Network, Xiamen University, Xiamen, China; ^4^First Affiliated Hospital of Wenzhou Medical University, Wenzhou Medical University, Wenzhou, China; ^5^Department of Biomedical Science, University of California Riverside, Riverside, CA, United States; ^6^School of Computer Science and Software Engineering, University of Science and Technology Liaoning, Anshan, China

**Keywords:** single-cell RNA sequencing, virus detection, SARS-CoV-2, deep learning, imbalanced data

## Abstract

**Introduction:**

Single-cell RNA sequencing (scRNA-seq) is a powerful tool for understanding cellular heterogeneity and identifying cell types in virus-related research. However, direct identification of SARS-CoV-2-infected cells at the single-cell level remains challenging, hindering the understanding of viral pathogenesis and the development of effective treatments.

**Methods:**

In this study, we propose a deep learning framework, the single-cell virus detection network (scVDN), to predict the infection status of single cells. The scVDN is trained on scRNA-seq data from multiple nasal swab samples obtained from several contributors with varying cell types. To objectively evaluate scVDN’s performance, we establish a model evaluation framework suitable for real experimental data.

**Results and Discussion:**

Our results demonstrate that scVDN outperforms four state-of-the-art machine learning models in identifying SARS-CoV-2-infected cells, even with extremely imbalanced labels in real data. Specifically, scVDN achieves a perfect AUC score of 1 in four cell types. Our findings have important implications for advancing virus research and improving public health by enabling the identification of virus-infected cells at the single-cell level, which is critical for diagnosing and treating viral infections. The scVDN framework can be applied to other single-cell virus-related studies, and we make all source code and datasets publicly available on GitHub at https://github.com/studentiz/scvdn.

## 1. Introduction

Single-cell RNA sequencing (scRNA-seq) has enabled the analysis of gene expression at the level of individual cells, opening up new avenues for understanding cellular heterogeneity and identifying rare cell types (Hu et al., 2022; Heumos et al., 2023). However, the analysis of scRNA-seq data is complicated by the presence of confounding factors such as batch effects (Zhang et al., 2022), cell cycle effects (Chang et al., 2015), and technical noise (Chai, 2022). In addition, the detection of viral infection in single cells is a challenging problem that has not been fully addressed (Luo et al., 2020). Among the numerous viral infections, SARS-CoV-2 is one of the biggest crises facing mankind at present (Tian et al., 2022).

SARS-CoV-2 is a highly infectious virus that has caused a global pandemic, with millions of confirmed cases and hundreds of thousands of deaths (Hu et al., 2021; Li et al., 2022). The virus primarily targets the respiratory system (Tuttolomondo et al., 2022), causing severe acute respiratory syndrome, potentially leading to respiratory failure and death (Li et al., 2021; Tabibzadeh et al., 2021). However, the virus can also infect other organs and tissues, including the heart (González-Calle et al., 2022), liver (Luo et al., 2022), and kidneys (Kadam et al., 2021). The cellular response to SARS-CoV-2 infection is complex and varies depending on the cell type and the severity of infection (Lamers and Haagmans, 2022), with studies revealing changes in gene expression and cell type composition as well as the activation of immune responses (Primorac et al., 2022; Hu et al., 2023a). However, the detection of infected cells has remained challenging, and existing methods are limited by low sensitivity and specificity (Harikrishnan et al., 2022).

To address this problem, we have developed a computational model called Single cell virus detection network (scVDN) to predict which cells are susceptible to virus infection. Our model takes scRNA-seq data as input and outputs a probability score for each cell, indicating the likelihood of viral infection. By identifying infected cells, our model can help to better understand the cellular response to viral infection.

To objectively evaluate the ability of the scVDN model, we compared its performance with four different machine learning models using the same training and testing datasets. Our results demonstrate that the scVDN model outperforms the other models in all tested cell types, with higher accuracy and lower misclassification rates. Our study reveals that the scVDN model has superior ability to accurately identify SARS-CoV-2-infected cell types compared to the other four models, indicating that scVDN shows higher precision and reliability in analyzing scRNA-seq data. The scVDN model utilizes deep learning methods to extract more discriminative features from gene expression data, which enables more accurate identification of infected cell types. Our research findings suggest that scVDN is more suitable for analyzing complex biomedical data compared to traditional machine learning models.

## 2. Materials and methods

### 2.1. Dataset

Single cell data for this study were obtained from the Jose Ordovas-Montanes team study (Ziegler et al., 2021). They collected viable cells using standard nasopharyngeal swabs and performed scRNA-seq, while host and viral RNA were analyzed. Specifically, nasal epithelial samples from 58 contributors were collected for a total of 32,588 cells, with 32,871 genes measured simultaneously per cell. We directly modeled the cellular data after preprocessing using Jose Ordovas-Montanes et al. including preprocessed gene expression, highly variable genes, annotated cell subtypes, annotated cell status (infected with SARS-CoV-2 or not), etc. They classified these samples into “COVID19_WHO_1–5,” “COVID19_WHO_6–8,” “Control_WHO_0” and “Control_WHO_8” based on WHO cohort scores. Among them, “COVID19_WHO_1–5” and “COVID19_WHO_6–8” contained cells infected by SARS-CoV-2, and we only applied these cell data for modeling. Specifically, “COVID19_WHO_1–5” was used as the training dataset (5,164 cells) and “COVID19_WHO_6–8” as the test dataset (12,909 cells) to evaluate the model performance. All datasets can be accessed at scVDN’s Github repository.

### 2.2. Siamese network

scVDN is a Siamese Network (Koch et al., 2015), which is a deep neural network structure for comparing similarities between two inputs. Siamese Network consists of two identical sub-networks, each with the same weights and structure, which share the same parameters. These two sub-networks pass the input data into their respective layers and generate two vector representations. These vector representations can be the features extracted at different layers or the outputs generated in the whole network.

Next, these two vector representations can be compared through the comparison layer, usually by computing one of the distance measures such as Euclidean distance (Chicco, 2021; Sun et al., 2022), cosine similarity (Berlemont et al., 2018; Hu et al., 2023b) or Manhattan distance (Imtiaz et al., 2020) between them to calculate the similarity or distance between them. This can be used for many tasks, such as face recognition, speech recognition, text similarity and recommender systems.

The structure of a twin network usually consists of the following components:

#### 2.2.1. Input layer

Two identical input networks, each receiving one input sample. For this study, the input layer requires the input of 3,508 highly variable genes per cell, which are derived from the study of Jose Ordovas-Montanes et al.

#### 2.2.2. Shared layers

Two identical subnetworks, each consisting of multiple layers that have the same structure and weights. These layers are used to extract features of the input data and generate a representation of the input vector (Woodbridge et al., 2018). These layers can be convolutional layers, pooling layers, fully connected layers or recurrent neural networks, etc. (Chung et al., 2017). In this study, the shared layer is a five-layer neural network structure. The first layer of the shared layer is a Dropout layer, which is used to cope with the sparsity challenge of scRNA-Seq (Jin et al., 2020). The second layer is a 784-dimensional neural network layer that extracts features from single-cell data. The third layer is a Layer Normalization, which has been shown to be effective in preventing model overfitting and further enhancing the generalization ability of the model (Xu et al., 2019). The fourth layer is a 256-dimensional neural network layer, which is used to extract features from single-cell data. The fifth layer is a 32-dimensional neural network layer. It is worth noting that the activation function of the fifth layer is “relu” (Li and Yuan, 2017; Wang et al., 2023), which makes the output of the shared layer always positive, which is crucial.

#### 2.2.3. Comparison layer

This layer is used to compare the similarity or distance of two vectors. The comparison layer can be any kind of function that is suitable for comparing two vectors. We choose cosine similarity as the comparison function. Since the output of the shared layer is always positive, this makes the value domain of the cosine function to be [0,1]. Where the output 0 of the comparison layer means, the two feature vectors are completely orthogonal (unrelated) and the output 1 means the two feature vectors overlap. This is a very critical optimization trick. Assuming that the output of the shared layer is not constrained, the cosine function has a value domain of [−1,1], where −1 means that the two feature vectors are unrelated and 1 means that the two feature vectors overlap. This can result in the failure of the model to converge.

#### 2.2.4. Output layer

The output layer takes the results of the comparison layer as input and outputs the final prediction of the network. For example, in a face recognition task, the output layer could be a binary classifier that predicts whether two input images are from the same person. In single-cell virus studies, the output layer outputs whether the features representing the two cell states (the state of being infected with SARS-CoV-2) are the same.

During training, the two input networks of the twin network receive the inputs simultaneously, and then extract features and generate vector representations through the shared layer. These vector representations are sent to the comparison layer for comparison, and then the output layer takes the comparison results as input for training.

During testing, the two input networks of the twin network receive two input samples and then the output layer outputs a prediction of whether they are similar or not. This network structure is commonly used for many tasks, such as face recognition, speech recognition, text similarity, and recommender systems.

### 2.3. Model dataset format

The data requirements for scVDN are consistent with those of Siamese Network, which is typically used for binary classification problems where two input samples need to be compared for similarity and to predict whether they belong to the same class. When training and testing the Siamese Network, the labels are set slightly differently.

When training the Siamese Network, the label is usually set to 0 or 1, indicating whether the two input samples belong to the same class. To achieve this label setting, pairs of training samples are used, and each training sample contains two input samples and their labels. Specifically, the two virus-infected cell pairs are labeled as 1. The two non-virus-infected cell pairs are also labeled as 1. The two cells are labeled as 0 when their virus-infection status is different.

Labeling is usually not necessary when testing the Siamese Network. Instead, the similarity score of the model output is used for classification. If the similarity score is greater than a certain threshold, the two input samples are determined to belong to the same class, otherwise they are determined not to belong to the same class. The choice of the threshold can be determined by tuning the performance of the model. In this study, the threshold value was set to 0.5.

It is important to note that the selection of sample pairs has a significant impact on the performance of the model when training and testing Siamese Network. Representative sample pairs need to be selected, and the number of positive and negative examples needs to be balanced to avoid bias in the model. Considering these factors, we choose ciliated cells as the modeling dataset. Specifically, we used the ciliated cells from the “COVID19_WHO_1–5” sample as training, with a total of 1,614 ciliated cells. Of these, 1,172 cells were labeled as negative (not infected by the virus), 100 cells were labeled as positive (infected by the virus), and 100 cells were labeled as ambiguous (unable to determine whether they were infected by the virus). We applied only positive and negative data for modeling. To balance the dataset, we sampled 2000 pairs of cell data from the positive dataset without duplication, and the labels of these pairs were recorded as 1. Similarly, we sampled 2000 pairs of cell data from the negative dataset without duplication, and the labels of these pairs were also labeled as 1. Finally, we sampled 4,000 pairs of cell data from the negative and positive datasets without duplication, and they were labeled as 0. Together, these data formed the training set for scVDN, at which point the ratio of labeled 1 to 0 was 1:1.

### 2.4. Model prediction stage

The application of scVDN to predict whether a cell is infected with a virus requires two inputs. One input is a single-cell dataset with known virus infection status, which is referred to as the reference dataset. The other input is, the single-cell dataset with unknown virus infection status, which is referred to as the query dataset. In this study, the virally infected single-cell data is the focus, so we use the virally infected single-cell data as the reference dataset. For each single-cell data to be predicted to be virally infected, we need to compare it with the whole reference dataset to determine whether it is virally infected or not. This comparison process is done by scVDN.

The output of scVDN is a vector that represents the similarity between two inputs with values in the range of 0 to 1. In this application, we consider it as a binary classification problem. By choosing a threshold value, say 0.5, we can convert the values in the output vector to 0 or 1. Specifically, an output greater than 0.5 is marked as 1, indicating that the cellular data to be queried is infected by the virus, while an output less than 0.5 is marked as 0, indicating that the cellular data to be queried is not infected by the virus.

To further improve the performance of the model, we used an integration strategy, namely the average scoring strategy. Specifically, for each query data, we compare it with each data in the reference dataset and average all the similarity scores as the predicted score for that query data. This prediction score can be used to determine whether the query data is virus-infected or not. With this integration strategy, we can reduce the overfitting of the model and improve the generalization ability and robustness of the model.

It should be noted that when applying scVDN for single-cell virus infection detection, it is necessary to ensure data quality, proper data preprocessing, selection of the appropriate Siamese Network, and a suitable model training strategy.

### 2.5. Evaluation indicators

Performance metrics, known as evaluation indicators, are used to measure the effectiveness and suitability of a model. In binary classification problems, commonly used evaluation indicators include accuracy (ACC), precision (PRE), recall (REC), F1 score (F1), receiver operating characteristic (ROC) curve and area under the curve (AUC). In the following section, we will introduce these evaluation indicators one by one.

ACC refers to the proportion of correctly classified samples to the total number of samples (Fair, 1986). The definition of ACC is shown in Eq. 1.


(1) $$ACC=\frac{TP+TN}{TP+FP+TN+FN}$$

In Eq. 1, TP represents the number of correct positive case predictions, FP is the number of incorrect negative case predictions, TN is the number of correct negative case predictions, and FN represents the data of incorrect positive case predictions.

ACC is usually used to evaluate the overall performance of a model, but it may be biased in imbalanced datasets. In this problem, the number of positive and negative virus cells is extremely imbalanced, with only a small portion of cells being classified as positive. Therefore, we do not consider accuracy as a reliable reference metric for this problem.

When dealing with imbalanced datasets, the number of positive and negative samples can be significantly different. If traditional accuracy metric is used to evaluate classifier performance, it may be biased towards the majority class. For example, in a dataset where the positive samples account for only 1% of the total samples and the negative samples account for 99%, if the classifier predicts all samples as negative, the accuracy will be 99%, but the classifier has not identified any positive samples. In this case, the accuracy cannot reflect the performance of the classifier, as the classifier has failed to recognize the target class, i.e., the positive samples. Balanced Accuracy (Balanced ACC) is a more appropriate evaluation metric for imbalanced datasets, as it takes into account the proportion of positive and negative samples and provides a more balanced assessment of classifier performance. Specifically, Balanced ACC calculates the average of the true positive rate (TPR) and true negative rate (TNR), which better reflects the performance of classifiers on imbalanced datasets. Therefore, compared to accuracy, Balanced ACC is more suitable for evaluating classifier performance on imbalanced datasets, as it can more accurately reflect the performance of classifiers and avoid evaluation bias caused by imbalanced samples.

PRE is the proportion of true positive samples to all samples predicted as positive by the model (Buckland and Gey, 1994). PRE is usually used to evaluate the prediction accuracy of a model, especially when the number of positive samples is small. The definition of PRE is shown in Eq. 2.


(2) $$PRE=\frac{TP}{TP+FP}$$

REC is the proportion of true positive samples to all actual positive samples (Allen and Casbergue, 1997). REC is usually used to evaluate the prediction ability of a model, especially when the number of positive samples is large. The definition of PRE is shown in Eq. 3.


(3) $$REC=\frac{TP}{TP+FN}$$

F1 is the harmonic mean of precision and recall, which can comprehensively consider the prediction accuracy and prediction ability of a model (Chicco and Jurman, 2020). The definition of PRE is shown in Eq. 4.


(4) $$F1=2*\frac{PRE*REC}{PRE+REC}$$

F1 takes into account both PRE and REC. The value of F1 ranges from 0 to 1, with a higher score indicating better model performance.

ROC curve is a curve plotted with recall as the y-axis and 1-precision as the x-axis, which can be used to measure the classification ability of a model (Hajian-Tilaki, 2013). AUC is the area under the ROC curve, which is usually used to evaluate the performance of a model (Bradley, 1997). The value of AUC ranges from 0 to 1, with a higher score indicating better classification ability of the model.

### 2.6. State-of-the-art models

We compare our scVDN model to four machine learning models with fundamentally different principles: k-Nearest Neighbors (KNN), Support Vector Machine (SVM), Naive Bayes (NB), and Random Forests (RF). All the machine learning models used in this study are obtained from the sklearn Python package (Feurer et al., 2022), which is a powerful scientific computing package that includes many high-performance machine learning models. The parameters of KNN, SVM, NB, and RF were set to their default values.

Our scVDN model is designed specifically for analyzing single-cell virus data, and it uses deep neural networks to learn high-dimensional features for virus classification. By comparison, KNN is a non-parametric classification model that classifies a sample based on the majority class of its k-nearest neighbors (Sun and Huang, 2010). SVM is a supervised learning model that separates different classes by finding the optimal hyperplane in a high-dimensional feature space (Hearst et al., 1998). NB is a probabilistic classification model that assumes the independence of features and calculates the probability of a sample belonging to a certain class based on its feature values (Jiang et al., 2008). RF is an ensemble learning model that combines multiple decision trees to improve the classification accuracy and stability (Genuer et al., 2010).

Overall, the choice of these models represents a diverse range of machine learning approaches. By comparing the scVDN model with these models, we demonstrate the effectiveness and superiority of our proposed approach for single-cell virus data analysis.

### 2.7. Open source and tutorials

We sought for authenticity and reliability. To ensure transparency, we have made all source code and data related to this research publicly available on our Github repository. Researchers can access not only the code used to generate the figures in this paper but also a series of tutorials we have prepared to aid in reproducing our work. We conducted this study entirely on a Google colab server (Carneiro et al., 2018), utilizing an NVIDIA Tesla V100 GPU to train our scVDN model. Additionally, we tested the performance of scVDN on a non-GPU server with only 50GB of memory. All models used in this study have been made available as pre-trained binaries on our Github repository, enabling users to call these models directly on a Google colab server. Overall, we hope our work will assist biomedical researchers in conducting more comprehensive single-cell virus studies.

## 3. Results

### 3.1. The workflow of scVDN model

**Figure 1:**
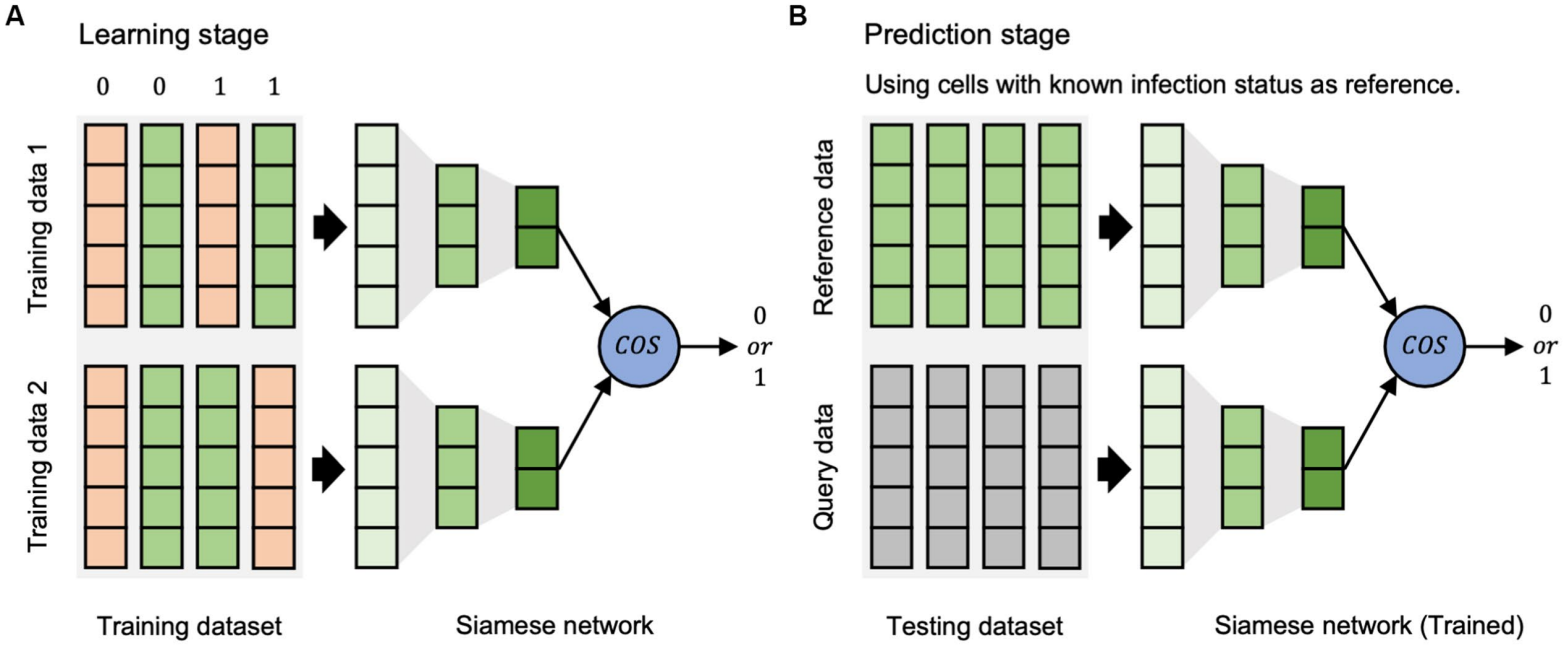
The workflow of scVDN model. **(A)** The learning stage is used to learn the differences between cell states. **(B)** The prediction stage is based on the reference dataset to determine the state of cells.

The scVDN model is a Siamese neural network that uses a pair of identical sub-networks to extract feature representations from pairs of input data points. The scVDN model consists of two stages: the learning stage (Figure 1A) and the prediction stage (Figure 1B).

In the learning phase, the scVDN model is trained on a labeled dataset, where each data pair is assigned a binary label (0 or 1) based on the same or different infection status between the two cells in the pair. The labels are designed to reflect the expected output of the scVDN model, which is to output a small distance between similar pairs (both infected or both uninfected) and a large distance between dissimilar pairs (one infected and one uninfected). For example, a data pair consisting of two cells infected with the virus would have a label of 1, while a pair of uninfected cells would also have a label of 1. A pair consisting of an infected and an uninfected cell would have a label of 0. The scVDN model utilizes a Siamese neural network architecture to extract feature representations from pairs of input data points, which we refer to as data pairs. The feature representations are then compared using cosine similarity to measure the similarity between the two input data points. The scVDN model learns the distance metrics between infected and uninfected cells by minimizing a contrastive loss function that encourages the distances between similar pairs to be small and the distances between dissimilar pairs to be large. The learned distance metrics capture the molecular differences between infected and uninfected cells, providing insights into the underlying mechanisms of viral pathogenesis and host response.

During the prediction stage, the scVDN model utilizes the learned distance metrics to identify SARS-CoV-2-infected cells within single-cell RNA sequencing data. For each unknown cell, also referred to as the query data, the scVDN model computes its pairwise distance to all infected cells in the reference dataset, also known as the reference data. This reference dataset comprises cells that are known to be infected with SARS-CoV-2 and serves as a positive control for identifying infected cells in the query data. The pairwise distances are calculated using cosine similarity between the feature representations of the query data and the reference data. Subsequently, the scVDN model assigns a similarity score to the query data based on the pairwise distances to the infected cells in the reference dataset, representing the likelihood that the query data contains SARS-CoV-2-infected cells. To calculate the final prediction score for the query data, the scVDN model computes the average of the similarity scores obtained for the query data with respect to all infected cells in the reference dataset. As the reference data solely comprises infected cells, the scVDN model can accurately identify SARS-CoV-2-infected cells in the query data. If the final prediction score exceeds a predefined threshold, typically set at 0.5, the query data is classified as infected with SARS-CoV-2. Conversely, if the final prediction score falls below the threshold, the query data is predicted to be uninfected.

In summary, the scVDN model is a siamese neural network that uses cosine similarity to measure the distance between embedded feature representations of input data points.

### 3.2. Performance evaluation framework for models

**Figure 2:**
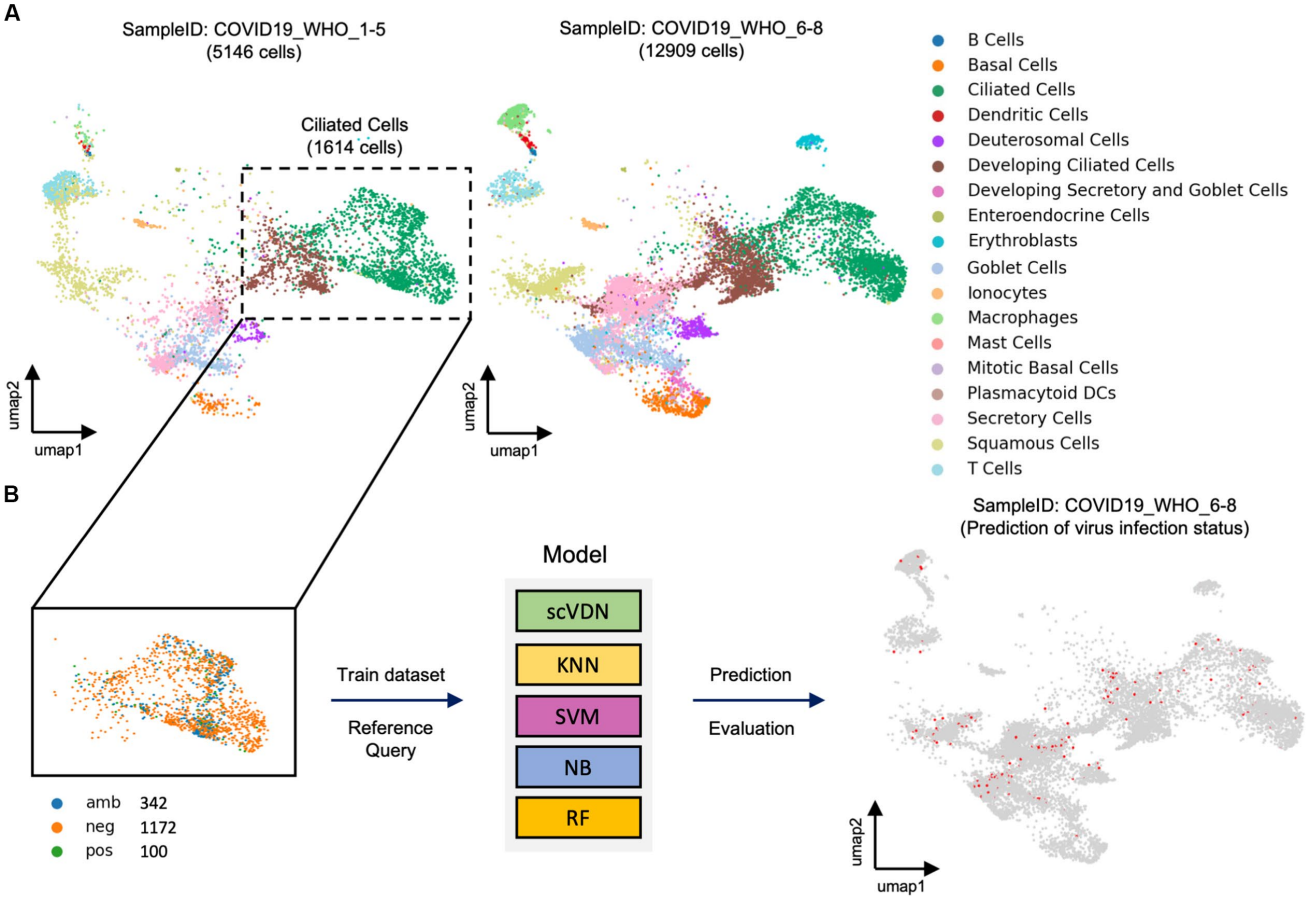
The evaluation framework for models designed for imbalanced real-world data. **(A)** Visualization of real-world single-cell data samples. **(B)** All models are trained using the same training dataset.

The task of virus detection at the single-cell level can be considered as a special binary classification problem. However, this is a highly challenging problem due to the extreme complexity and imbalance of real-world single-cell data. To evaluate the scVDN model’s ability to process real-world single-cell data, we constructed an objective evaluation system using a large cohort of SARS-CoV-2-positive nasal swab samples. We used cells labeled “COVID-19_WHO_1–5” as the training dataset and cells labeled “COVID-19_WHO_6–8” as the test dataset (Figure 2A). Notably, there were only 5,146 cells in the training dataset, while the test dataset contained 12,909 cells, making it a highly challenging modeling task.

The reason for using a test dataset with a larger sample size than the training dataset is to evaluate the generalization capability of the scVDN model. In other words, we wanted to assess how well the model can identify SARS-CoV-2-infected cells in new, unseen data that was not used during the training process. By testing the model on a larger and more diverse set of samples, we were able to better evaluate its performance and robustness. Moreover, using a test dataset with a larger sample size also helps to reduce the risk of overfitting, which occurs when a model is too complex and fits the training data too well, resulting in poor performance on new, unseen data. By testing the model on a larger dataset, we can better assess its ability to generalize to new data and avoid overfitting.

In the “COVID-19_WHO_1–5” cell subset, we observed that only 150 cells were labeled as infected with SARS-CoV-2, which is less than 1% of the total number of cells in the subset. This extreme class imbalance poses a challenge for traditional machine learning models to build a binary classification model. Further analysis revealed that 100 of the infected cells were ciliated cells. Therefore, we decided to use only ciliated cells as the training data for the scVDN model (Figure 2B). Among the ciliated cells, 100 were infected with SARS-CoV-2 (positive), 1,172 were uninfected (negative), and 342 cells had an ambiguous status. We removed the cells with ambiguous status and used only the cells labeled as positive or negative as training samples. By focusing on ciliated cells and using only the cells labeled as positive or negative, we were able to reduce the class imbalance and improve the model’s performance. This approach also allowed us to better train the model on the primary targets of SARS-CoV-2 infection and reduce the risk of false positives or false negatives on other cell types.

To provide a more objective evaluation of the scVDN model’s ability, we compared it with four different machine learning models with different underlying principles. These models were KNN, SVM, NB, and RF. All models were trained using the same training dataset (Figure 2B). To ensure that any performance differences between the scVDN model and the other models were solely due to differences in modeling principles, we restricted the reference data for the scVDN model to the training dataset only.

### 3.3. The performance of models

**Figure 3:**
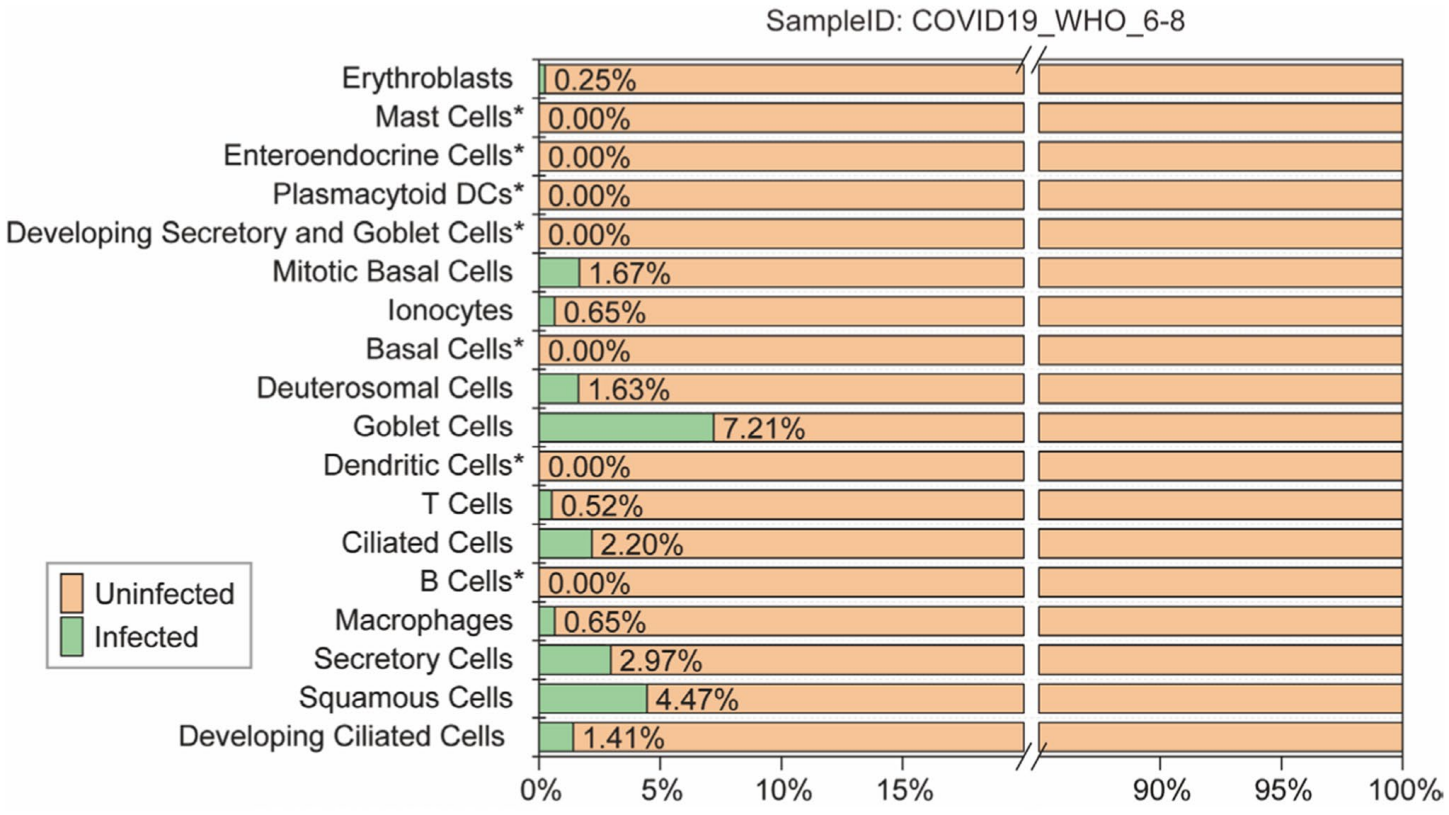
The ratio of infected and uninfected cells in each cell subtype of the “COVID-19_WHO_1–5” cell set is calculated for evaluating the models’ performance. Cell subtypes marked as “not included” do not participate in the model performance evaluation.

To accurately evaluate the performance of each model, all models were predicting each cell subtype within the “COVID-19_WHO_6–8” dataset. This dataset is comprised of 18 different cell subtypes, of which 11 contains both infected and uninfected cells. Only the predictions for these 11 cell subtypes were evaluated (Figure 3). The remaining 7 cell subtypes do not contain any infected cells and thus cannot be comprehensively evaluated using various performance metrics such as ACC and AUC. Therefore, the predictions for these 7 cell subtypes were not included in the evaluation.

**Figure 4:**
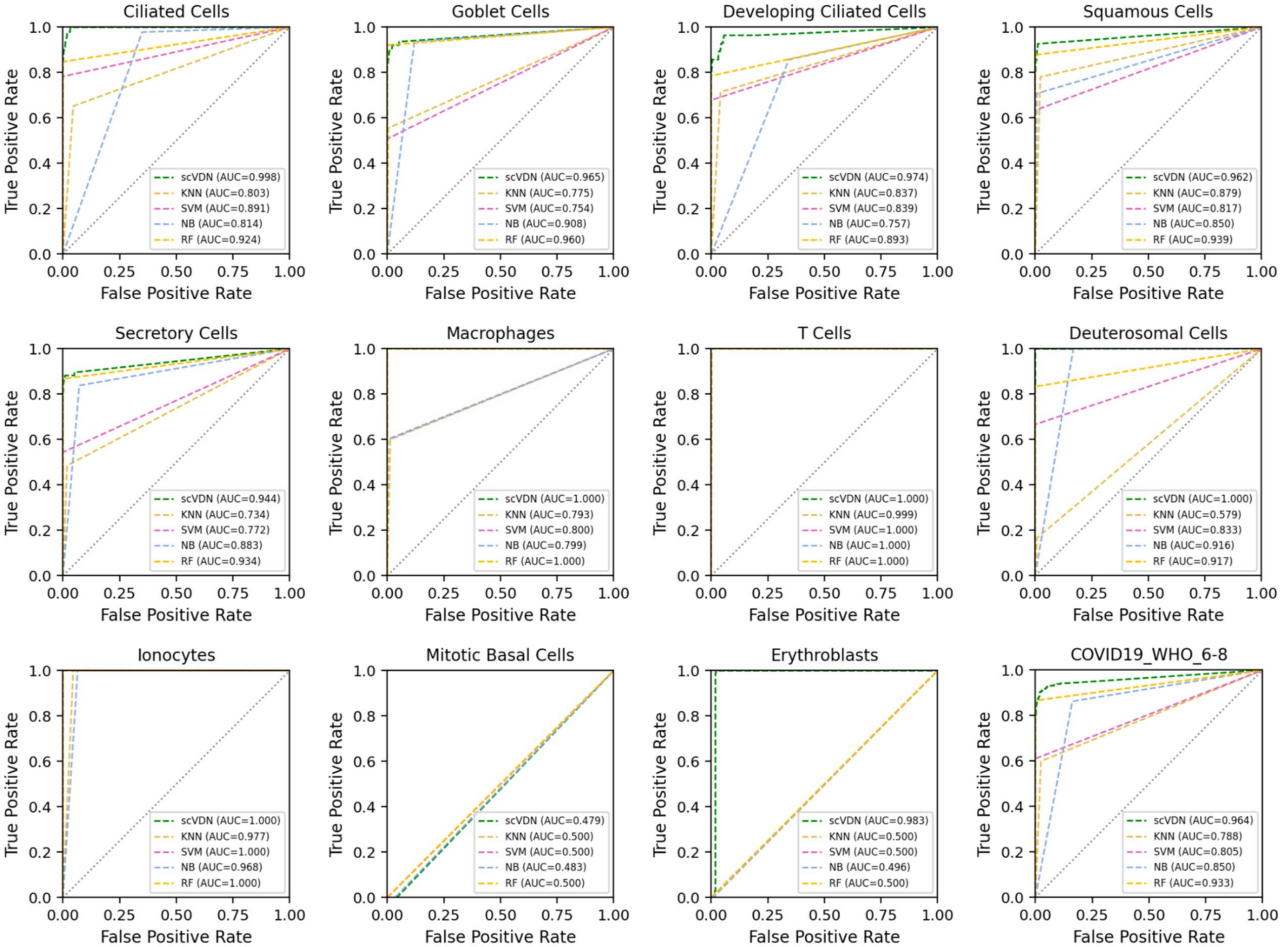
The ROC curves and AUC values of the prediction results for each cell subtype in the “COVID-19_WHO_6–8” dataset for all models.

We find that the scVDN model achieves the highest AUC scores in predicting almost all cell subtypes, including the entire “COVID-19_WHO_6–8” dataset without distinguishing between cell types (Figure 4). Remarkably, scVDN is only trained on ciliated cells, yet it accurately predicts the infection status of other cell types, demonstrating its strong generalization ability. The superior performance of scVDN in predicting infection in different subtypes of cells can be attributed to several factors. Firstly, ciliated cells may share certain features or signaling pathways with other cell subtypes that are relevant to viral infection, allowing scVDN to generalize its knowledge to other cell subtypes. Secondly, scVDN may have learned to capture common fundamental features or patterns of infected cells that are applicable to different cell subtypes. Lastly, scVDN may have effectively filtered out noise or irrelevant features in the training data, enabling it to focus on the critical features required for viral detection in different cell subtypes.

However, an important issue arises in that all models perform poorly in predicting the infection status of Mitotic Basal Cells, with AUC values hovering around 0.5. This indicates that the models’ performance in predicting the infection status of these cells is no better than random chance. This issue can be attributed to several factors. Firstly, Mitotic Basal Cells may possess unique features or signaling pathways that were not captured in the training data, making it challenging for the models to generalize their knowledge to these cells. Secondly, the training data may be insufficient or imbalanced in representing Mitotic Basal Cells, leading to poor performance in predicting their infection status. Lastly, technical issues may have arisen during data collection or labeling, resulting in incorrect or ambiguous labeling of Mitotic Basal Cells, which could have affected the models’ performance.

We conducted an in-depth analysis of the performance of all models in the COVID-19_WHO_6–8 dataset with respect to other evaluation metrics. We found that all models have very high ACC (Table 1). In binary classification models, accuracy is one of the most commonly used evaluation metrics, as it reflects the proportion of correctly predicted samples in the entire dataset. However, in cases of imbalanced sample labels, accuracy can be misleading. For instance, in this study, the number of positive samples is small, while the number of negative samples is large, and a model may predict all samples as negative to obtain high accuracy. In such cases, accuracy does not reflect the actual performance of the model.

**Table 1:** Evaluation metrics for all models on the “COVID-19_WHO_6–8” dataset.

Model	ACC	Balanced_ACC	PRE	REC	F1	AUC
scVDN	0.991	0.924	0.778	0.855	0.815	0.964
KNN	0.967	0.787	0.376	0.599	0.462	0.788
SVM	0.991	0.805	1.000	0.611	0.758	0.805
NB	0.838	0.850	0.115	0.863	0.202	0.850
RF	0.997	0.933	1.000	0.866	0.928	0.933

**Figure 5:**
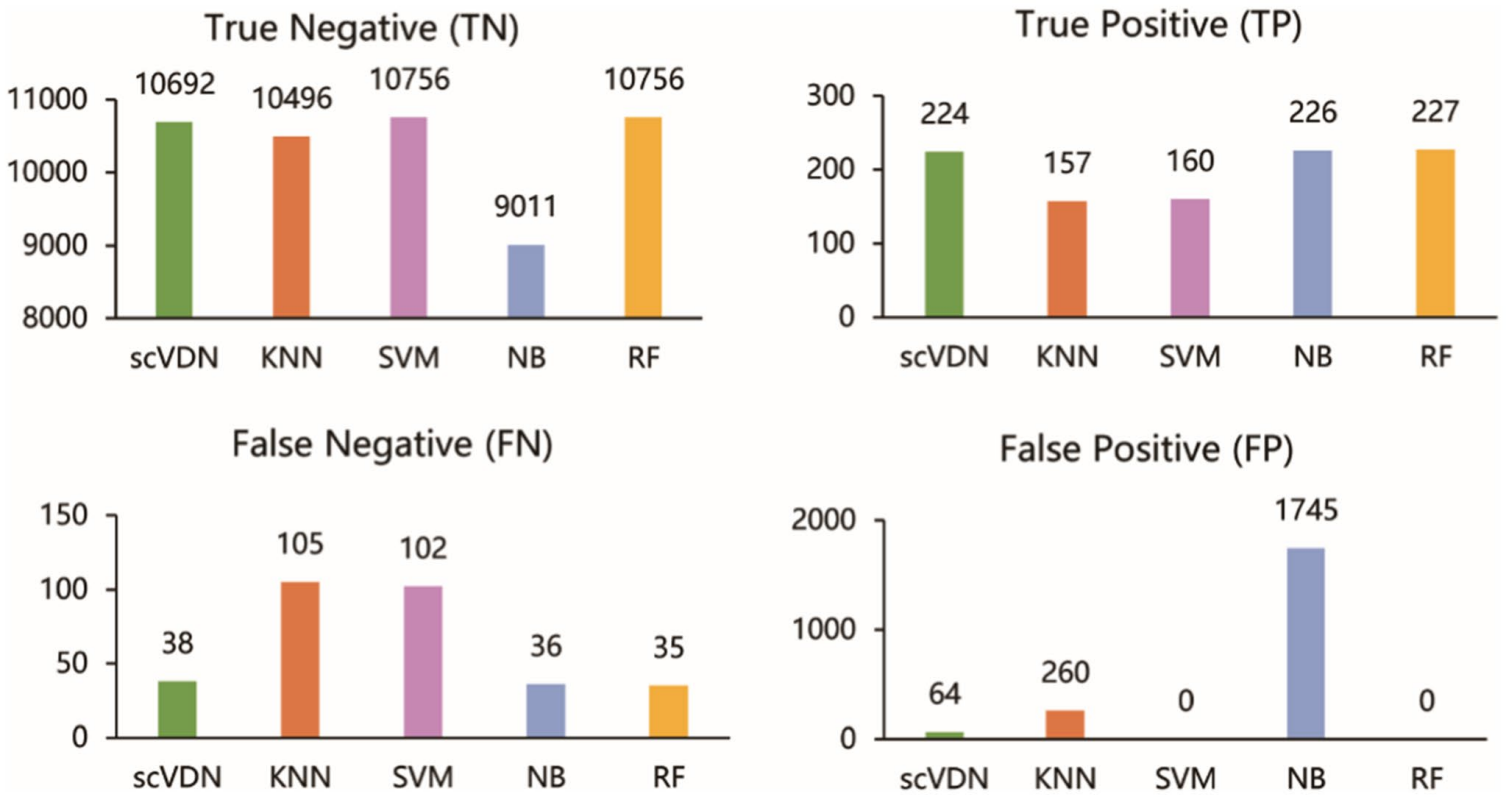
The evaluation of all models on the “COVID-19_WHO_6–8” dataset.

Moreover, we also noticed that the FP of SVM and RF models is 0 (Figure 5). This is not desirable in biomedical research on diseases. In many biomedical studies, we prefer models that tend to produce low levels of false positive results, as it helps researchers identify potential anomalies. For instance, in cancer diagnosis, if the diagnostic result cannot guarantee 100% accuracy (currently no model can achieve this), we would rather believe the positive diagnosis result than easily believe the negative diagnosis result. In virus infection studies, we pay more attention to the positive results of the model’s outputs, as it helps us detect viruses in the patient’s body and take corresponding treatment measures. In this case, the false positive rate of the model is more important. In addition, we found that all models were unable to accurately predict the virus infection status of Mitotic Basal Cells, which may indicate the presence of labeling errors in this part of the dataset. To ensure the quality of the dataset, stricter labeling and verification are needed in cases of inaccurate labeling. If labeling errors exist, they may affect the performance and practical value of the model. In such cases, we need to evaluate the model’s output results more carefully, especially the false positive rate. Considering all these factors, we believe that the scVDN model has stronger practical value than other models in virus infection studies.

## 4. Discussion and conclusion

In this study, we proposed a novel deep learning framework called scVDN, for predicting the infection status of single cells. Unlike classical binary classification models, scVDN does not directly predict whether a single cell is infected with a virus. Instead, it first learns a measure of the cell’s state and then predicts the infection status of unknown cells based on the reference of known infected and uninfected cells. To evaluate the predictive performance of scVDN on real-world data, we trained and tested the model on a single-cell sequencing dataset from a nasal swab cohort. However, the number of cells infected with the SARS-CoV-2 in the real dataset is much smaller than the number of uninfected cells, resulting in extreme data imbalance. To address this, we only used ciliated cells as the modeling data and predicted the infection status of cells in another nasal swab cohort. We also compared scVDN with state-of-the-art machine learning models and found that scVDN outperformed them in all test scenarios.

Our results demonstrate that scVDN is a promising framework for predicting the infection status of single cells. By learning a measure of the cell’s state, scVDN can effectively address the issue of data imbalance and improve the accuracy of prediction. The ciliated cells used in our study are known to be a primary target for respiratory viruses, including the SARS-CoV-2, which makes them a suitable choice for modeling data. Our findings suggest that scVDN can be extended to other datasets and cell types for predicting infection status.

scVDN is a type of Siamese Network that has been developed for the binary classification task of identifying whether a cell in single-cell data is infected with the SARS-CoV-2 virus, which is the virus responsible for causing the COVID-19 disease. Compared to traditional machine learning models, scVDN has several advantages. Firstly, scVDN is a neural network model that can automatically extract features from the input data, which is useful when the data is complex and high-dimensional. Secondly, scVDN is specifically designed to handle imbalanced and small datasets, which is often the case in virus infection detection. Thirdly, scVDN can learn from limited labeled data, making it suitable for cases where labeled data may be scarce or expensive to obtain. Finally, scVDN can capture complex relationships between inputs, which is useful in virus infection detection, where the relationship between infected and healthy cells may be complex and difficult to define explicitly. Overall, scVDN can be a powerful tool for binary classification tasks in single-cell data analysis, especially when the dataset is complex, imbalanced, and limited in size, and when the goal is to identify whether a cell is infected with the SARS-CoV-2 virus.

One potential application of scVDN is the diagnosis of viral infections. With the COVID-19 pandemic still ongoing, there is a pressing need for accurate and efficient diagnostic tools. Our study shows that scVDN can accurately predict the infection status of single cells, which could be useful for identifying infected individuals and tracking the spread of the virus.

Another potential application of scVDN is drug discovery. By predicting the infection status of single cells, scVDN can be used to identify potential drug targets and test the efficacy of candidate drugs. This could lead to the development of more effective treatments for viral infections.

Our study also has limitations that should be considered when interpreting the results. The dataset used in our study came from nasal swab cohort and may not be representative of other populations or disease states. Additionally, there is currently no way to guarantee that the known infected cells in the dataset are completely reliable, which can lead to false positives and potential risks. As such, future biomedical research should pay more attention to the label distribution of datasets and researchers should be more cautious in interpreting and applying the model’s predictions, especially in situations where the labels are not completely reliable. To address these limitations, future studies could use larger and more diverse datasets to validate the findings of this study. New technologies or methods could also be developed to improve the accuracy and reliability of single-cell sequencing data. We believe that these efforts will provide more accurate, reliable, and practical tools and methods for biomedical research and promote further development and progress in the field.

In addition to its potential for COVID-19 detection, scVDN has broad applicability for detecting other respiratory diseases, such as influenza and respiratory syncytial virus. This novel approach to predicting infection status has the potential to transform the field of biomedical research, providing researchers with a more accurate and efficient tool for studying infectious diseases. Our findings represent a significant advancement in the development of diagnostic tools with broad applications, ultimately improving health outcomes for patients worldwide.

## Data availability statement

The original contributions presented in the study are included in the article/supplementary material, further inquiries can be directed to the corresponding author.

## Author contributions

HH conducted the scRNA-seq experiments, developed and implemented the scVDN framework, and analyzed the data. ZF contributed to the development and implementation of the scVDN framework, performed data analysis, and helped draft the manuscript. XS contributed to the experimental design, development and implementation of the scVDN framework, and data analysis. JL contributed to the experimental design, development and implementation of the scVDN framework, and data analysis. XL and HL conceived and supervised the study, secured funding and resources, and provided critical insights and guidance throughout the project. JS provided technical support, contributed to the experimental design, and helped draft the manuscript. All authors critically reviewed and edited the manuscript.

## Funding

This work was supported by the Ministry of Science and Technology of the People's Republic of China (STI2030-Major Projects2021ZD0201900), the National Natural Science Foundation of China (Grant No. 12090052), Foundation of Education Department of Fujian Province Foundation (Grant No. 2020Y4001).

## Conflict of interest

The authors declare that the research was conducted in the absence of any commercial or financial relationships that could be construed as a potential conflict of interest.

## Publisher’s note

All claims expressed in this article are solely those of the authors and do not necessarily represent those of their affiliated organizations, or those of the publisher, the editors and the reviewers. Any product that may be evaluated in this article, or claim that may be made by its manufacturer, is not guaranteed or endorsed by the publisher.
